# Neddylation pathway alleviates chronic pancreatitis by reducing HIF1α-CCL5-dependent macrophage infiltration

**DOI:** 10.1038/s41419-021-03549-3

**Published:** 2021-03-15

**Authors:** Yuli Lin, Yusheng Chen, Wenxue Feng, Rong Hua, Junfeng Zhang, Yanmiao Huo, Hong Jiang, Bo Yin, Xuguang Yang

**Affiliations:** 1 Clinical Research Center, Department of Oncology, Longhua Hospital, Shanghai University of Traditional Chinese Medicine, Shanghai, China; 2 Department of Immunology, School of Basic Medical Sciences, Fudan University, Shanghai, China; 3 Department of Pancreatic Surgery, Department of Oncology, Pancreatic Cancer Institute, Shanghai Cancer Center, Shanghai Medical College, Fudan University, Shanghai, China; 4 Department of Radiology, Huashan Hospital, Fudan University, Shanghai, China; 5 Department of Biliary-Pancreatic Surgery, Renji Hospital, School of Medicine, Shanghai Jiao Tong University, Shanghai, China; 6 Department of General Surgery, Huadong Hospital, Fudan University, Shanghai, China

**Keywords:** Neddylation, Neddylation, Pancreatitis, Immunopathogenesis, Inflammation

## Abstract

Chronic pancreatitis (CP) is characterized by a wide range of irreversible fibro-inflammatory diseases with largely ambiguous pathogenesis. Although neddylation pathway has been implicated in regulating immune responses, whether the dysregulation of neddylation is involved in the progression of CP and how neddylation regulates the inflammatory microenvironment of CP have not yet been reported. Here, we demonstrate that global inactivation of neddylation pathway by MLN4924 significantly exacerbates chronic pancreatitis. The increased M2 macrophage infiltration, mediated by the upregulated chemokine (C-C motif) ligand 5 (CCL5), is responsible for the enhanced pancreatitis-promoting activity of MLN4924. Both CCL5 blockade and macrophage depletion contribute to alleviating pancreatic fibrosis and inflammation in MLN4924-treated CP mice. Mechanistic investigation identifies that inactivation of Cullin-RING ligases (CRLs) stabilizes cellular levels of hypoxia-inducible factor 1α (HIF-1α), which increases CCL5 expression by promoting CCL5 transactivation. Clinically, UBE2M expression remarkably decreases in human CP tissues compared with normal specimens and the levels of *CCL5* and M2 marker *CD163* are negatively correlated with UBE2M intensity, suggesting that neddylation is involved in the pathogenesis of pancreatitis. Hence, our studies reveal a neddylation-associated immunopathogenesis of chronic pancreatitis and provide new ideas for the disease treatment.

## Introduction

Chronic pancreatitis (CP) is defined as a progressive and irreversible fibro-inflammatory syndrome of the pancreas and eventually leads to the damage of pancreatic function^1^. The environmental (alcohol and cigarettes) and genetic factors are key risk factors of CP^2–4^. Histologic characteristics of CP include inflammatory infiltrates, fibrosis, acinar cell atrophy, duct distortion as well as squamous metaplasia of the duct epithelium^5,6^. Currently, neither “gold standard” diagnostic test nor active therapeutic agent exists for CP and most patients remain symptomatic after limited supportive management^6,7^.

The mechanisms and pathways to CP are still complicated and ambiguous. CP mostly begins in recurrent episodes of acute inflammatory bouts of the pancreas parenchyma, followed by activating pancreatic stellate cells and initiating pancreatic fibrogenesis^7^. A well-accepted hypothesis regarding the pathogenesis of chronic pancreatitis is that pancreatic inflammation is initiated by inflammatory immune microenvironment. A complex mix of acinar cells, stromal cells (e.g., immunocytes and fibroblast cells) as well as soluble factors interplay with each other and make up the inflammatory microenvironment of CP^8^. Among the immune cells infiltrating in the microenvironment of CP, myeloid cells especially macrophages play an important role in regulating the progression of the disease. Earlier studies have confirmed that macrophages are the predominant myeloid cell infiltrates in CP^9–11^. Macrophages are characterized by marked phenotypic heterogeneity, as a result of cellular differentiation. In response to various signals, macrophages are divided into two types: classically activated macrophages (M1) and alternatively activated macrophages (M2). M1 play a critical role in host defense and anti-tumor immunity, and M2 are involved in tissue remodeling, fibrosis and tumor progression^12–15^. The pancreatic environment can tame the type and functional characteristic of macrophages present in CP. Studies have shown pancreatic stellate cells (PSCs)-derived IL-4 signal contributes to M2 polarization in CP^11,16,17^. PSCs are myofibroblast-like cells in the pancreas, characterized by the expression of alpha-smooth muscle actin (α-SMA) and extracellular-matrix (ECM) proteins upon activated by cytokines released from acinar cells and/or leukocytes. Activated macrophages potently activate fibroblasts by transforming growth factor-beta (TGFβ) or other cytokines and induce ECM synthesis, which ultimately promote the tissue damage and fibrosis^8,18,19^. Despite the vital roles of macrophages in the pathogenesis of CP, the regulation of macrophage infiltration in CP remains to be defined.

Neddylation is a reversible post-translational modification that adding a ubiquitin-like molecule NEDD8 (neuronal precursor cell-expressed developmentally down-regulated protein 8) to the lysine residue in specific substrate proteins via a successive three-step enzymatic reaction, catalyzed by NEDD8-activating enzyme E1 (a heterodimer composed of NAE1 and UBA3), NEDD8-conjugating enzyme E2 (UBE2M or UBE2F), and substrate-specific NEDD8-E3 ligases. The cullin subunits of Cullin-RING-ligases (CRLs) are the largest family of multiunit E3 ubiquitin ligases as well as the main targets of NEDD8^20–22^. MLN4924, a small molecular inhibitor of neddylation activation, covalently adducts with NEDD8, competitively binds to the active site of NAEβ, and blocks the CRLs activation^23^. Importantly, some CRL substrates (e.g., IκB and HIF-1α) trigger multiple biological events in aspects of the proliferation, effector function and signal transduction of diverse immune cells, and are involved in the regulation of the immune system. Dysregulation of neddylation activation leads to abnormal degradation of CRL substrates, followed by ectopic immune responses in vitro and in vivo^24–27^. Even though neddylation has been established as a critical CRL-dependent mediator involved in the regulation of inflammation (e.g., colitis, atherosclerosis, acute lung injury)^27–29^, whether neddylation affects the process of chronic pancreatitis has not been mentioned. Herein, we aim to clarify the effects and underlying mechanisms of neddylation in the inflammatory microenvironment of CP.

In this study, we demonstrate that inactivation of neddylation is associated with the immunopathogenesis of CP, which orchestrates the inflammatory microenvironment with M2 macrophage infiltration. Notably, neddylation-regulated macrophage infiltration is dependent on the HIF-1α-CCL5 axis, indicating a crosstalk between macrophages and acinar cells in inflammatory pancreata. These findings are likely to partly delineate immune responses involved in such fibro-inflammatory disease, which improves our understanding of the pathogenesis and offers potential benefits for the disease treatment.

## Results

### Neddylation inactivation by MLN4924 exacerbates chronic pancreatitis

**Fig. 1:**
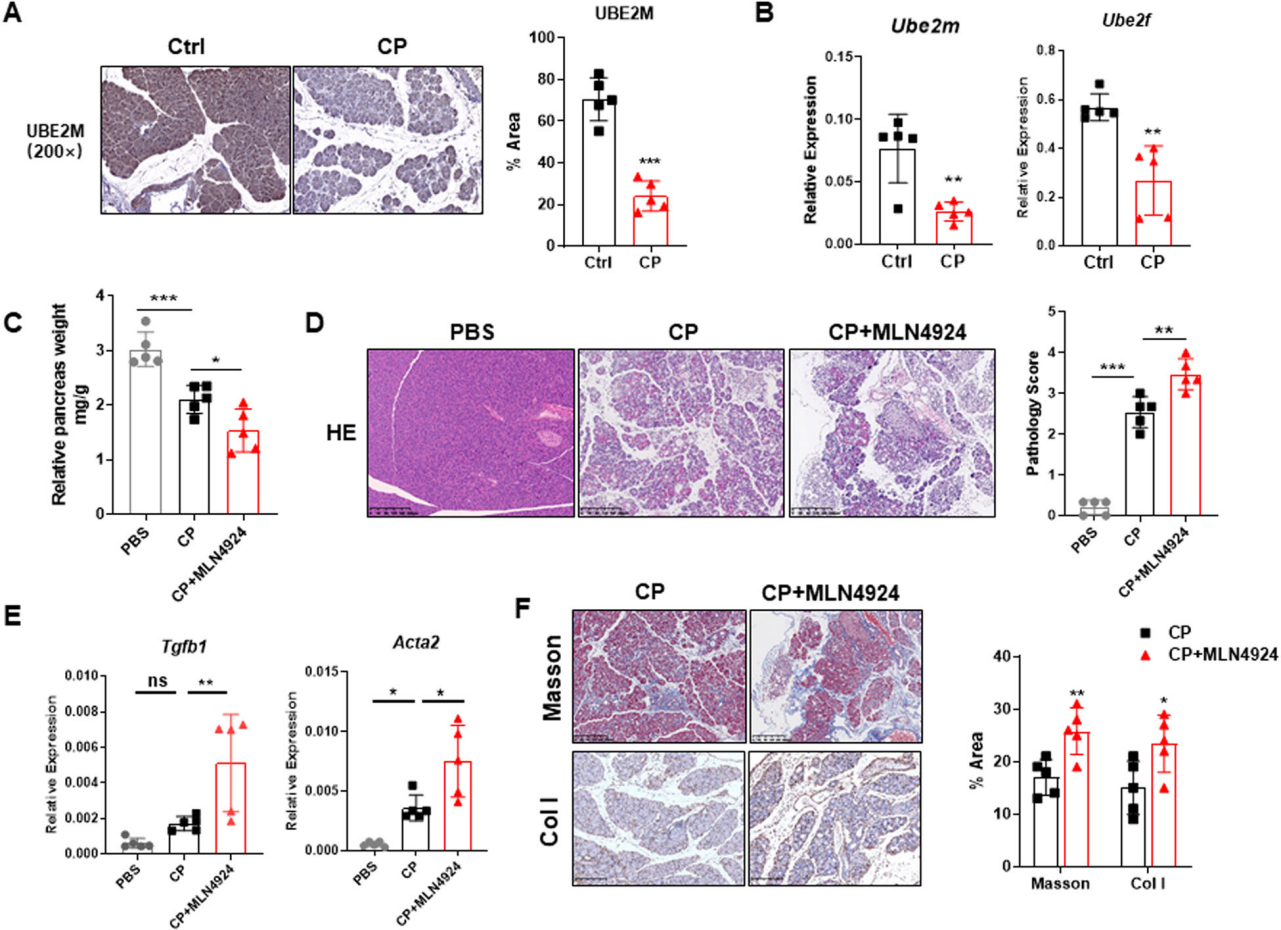
Neddylation inactivation exacerbates chronic pancreatitis. **A** Representative IHC images of UBE2M in normal and CP pancreata. **B** mRNA expression of *Ube2m* and *Ube2f*. **C** Relative pancreas weight (pancreas weight/body weight). **D** Representative H&E images and pathological scores. Scale bar, 200 μm. **E** mRNA expression of *Tgfβ* and *Acta2* (αSMA). **F** Representative Masson images, IHC images of collagen I, and bar graphs. Scale bar, 200 μm. **A**–**F** *n* = 5. Data are representative of three independent experiments. *p < 0.05, **p < 0.01, ***p < 0.001, ns no significant.

Herein, we sought to investigate the effect of neddylation pathway on the immunopathogenesis of chronic pancreatitis. For this purpose, we established a model of chronic pancreatitis (CP) through repeated injection of mice with caerulein. We first evaluated the expression of the NEDD8-conjugating enzymes UBE2M and UBE2F by immunohistochemistry and RT-PCR and found decreased levels of UBE2M and UBE2F in pancreatic tissues from CP mice compared to control mice (Fig. 1A, B). Mice administered the CP regimen clearly manifested glandular atrophy, infiltration of immune cells and distorted and/or blocked ducts. Global inactivation of neddylation pathway by MLN4924 exacerbated chronic pancreatitis, as evidenced by much smaller pancreas size and obvious histopathological features (Fig. 1C, D). With respect to pancreatic fibrosis, we corroborated higher fibrosis-associated gene expression, such as *Tgfβ* and *Acta2* (αSMA), in the pancreas from CP mice administrated with MLN4924 (Fig. 1E). Additionally, inactivation of neddylation pathway in CP mice demonstrated increased PSC activation and collagen deposition, as shown by Masson trichrome gelatin staining and immunohistochemical analysis of collagenI (Fig. 1F). Altogether, these data indicate that normal pancreas have higher expression of neddylation pathway and neddylation protects against caerulein-induced pancreatic injuries.

### Neddylation inactivation contributes to elevated M2 macrophage infiltration

**Fig. 2:**
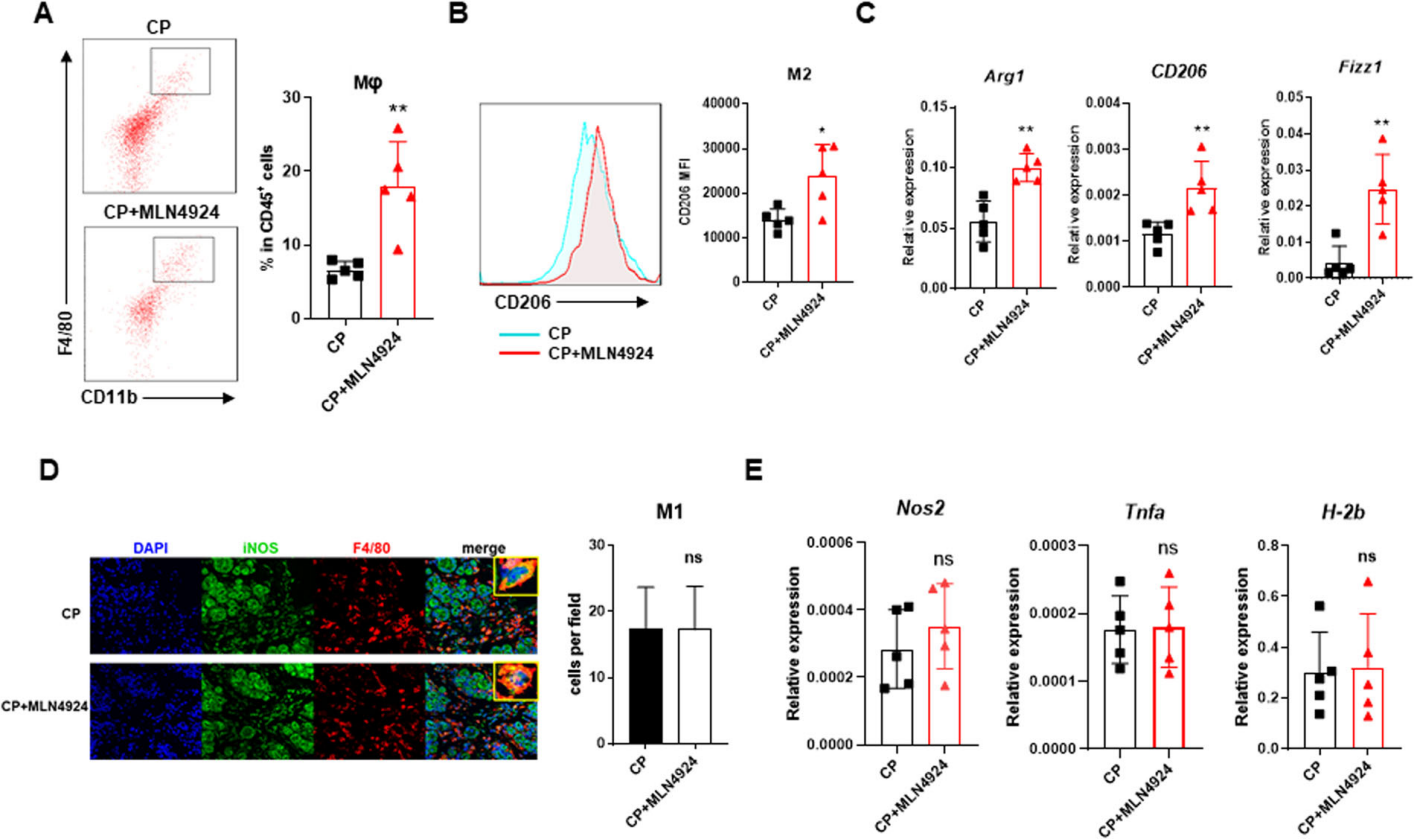
Neddylation inactivation contributes to elevated M2 macrophage infiltration. **A** Representative flow cytometric figures and percentages of macrophages in pancreata from different groups of mice. **B** Representative flow cytometric figure and mean fluorescence intensity of CD206 in pancreatic macrophages. **C** mRNA expression of M2-associated genes *Arg1*, Fizz1 and CD206. **D** Representative immunofluorescence images of pancreata, co-stained with F4/80, iNOS and DAPI. **E** mRNA expression of *Nos2, Tnfa*, and *H-2b* in pancreata. **A**–**E** *n* = 5. Data are representative of three independent experiments. *p < 0.05, **p < 0.01, ***p < 0.001, ns no significant, MFI mean fluorescence intensity.

To interrogate the cellular basis in the inflammatory microenvironment by which MLN4924 promotes chronic pancreatitis, we examined the infiltration of total T cells, T-cell subsets (including CD4^+^ T cells, CD8^+^ T cells, Th17 cells and Treg cells) and NK cells, and found that there were no differences in frequencies of these cells between MLN4924-treated CP tissues and control CP tissues, which suggesting that neddylation might not affect the infiltration of these cells in CP (Fig. S1A–C). Then we detected the frequency of macrophages that proposed as the major regulators of inflammation and fibrosis. Results showed that MLN4924 treatment in CP mice promoted the infiltration of macrophages in pancreata (Fig. 2A). In view that alternatively activated macrophages are dominant in pancreata with CP^11^ and MLN4924 is indicated to drive macrophage polarization toward the M2 phenotype^28^, we further analyzed the expression of M1- and M2-associated markers. As expected, pancreatic macrophages isolated from MLN4924-treated CP mice had higher M2-marker CD206 compared with CP mice (Fig. 2B). Moreover, RT-PCR analysis exhibited alternative M2 profile with increased expression of *Arg1*, *CD206* and *Fizz1* after MLN4924 treatment (Fig. 2C), indicating the MLN4924-enhanced infiltration of M2. With regard to M1, no distinct discrepancy of iNOS^+^F4/80^+^ M1 infiltration was detected by immunofluorescence in CP mice and MLN4924-CP mice (Fig. 2D). The mRNA expression of *Nos2* confirmed the similar classical activation profile in pancreatic macrophages in CP mice and MLN4924-CP mice (Fig. 2E). Moreover, neddylation inactivation did not affect the mRNA expression of other M1-associated markers, such as major histocompatibility complex class II (*MHCII, H-2b*) and tumor-necrosis factor alpha (*Tnfa*) (Fig. 2E). Collectively, inactivation of neddylation pathway by MLN4924 promotes the infiltration of M2 macrophages, but not M1, in the inflammatory microenvironment of pancreata.

### Neddylation inactivation promotes pancreatitis in a macrophage-dependent manner

**Fig. 3:**
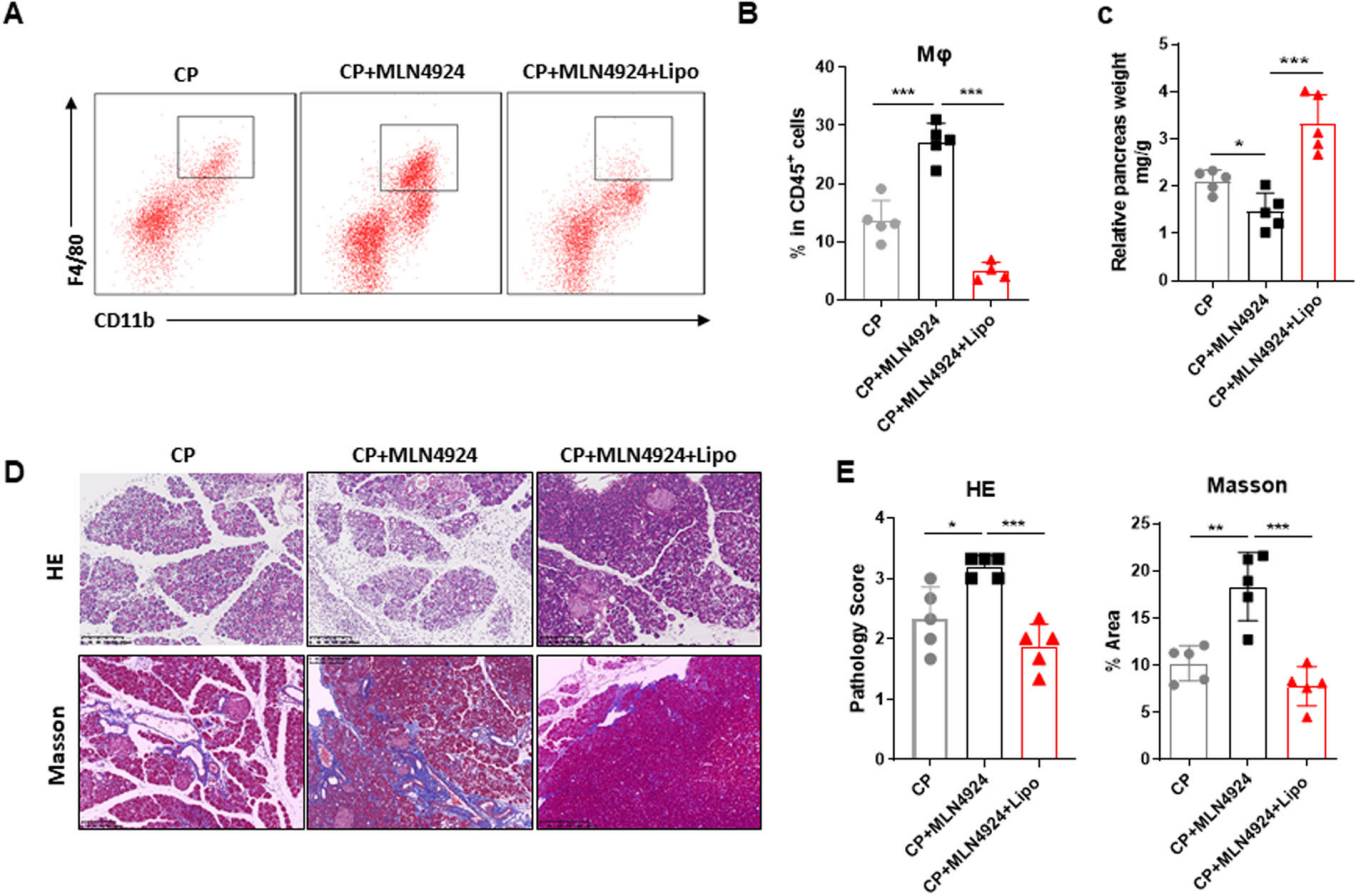
Neddylation inactivation promotes pancreatitis in a macrophage-dependent manner. **A**, **B** Representative flow cytometric figures and percentages of macrophages in pancreata from different groups of mice. **C** Relative pancreas weight (pancreas weight/body weight). **D** Representative H&E and Masson images of pancreata. Scale bar, 200 μm. **E** Bar graphs of **D**. **A**–**E** *n* = 5. Data are representative of three independent experiments. *p < 0.05, **p < 0.01, ***p < 0.001, ns no significant.

To demonstrate whether the promotion of neddylation inactivation in CP is macrophage dependent, we performed the macrophage depletion by intraperitoneal injection with clodronate liposomes. Treatment with clodronate liposomes was sufficient to decline the frequency of macrophages upregulated by MLN4924 (Fig. 3A, B), confirming the efficient depletion of macrophage in vivo. The larger pancreas size as well as relatively normal tissue architecture manifested alleviated chronic pancreatitis in liposome-treated mice (Fig. 3C–E). Masson trichrome gelatin staining of pancreata demonstrated that depletion of macrophages impaired the activation of pancreatic satellite cells, indicating slight pancreatic fibrosis (Fig. 3D, E). Taken together, these data prove that infiltrated macrophages in the pancreatic microenvironment play important roles in neddylation-associated immunopathogenesis of chronic pancreatitis.

### Neddylation inactivation elevates CCL5 secretion to recruit macrophages

**Fig. 4:**
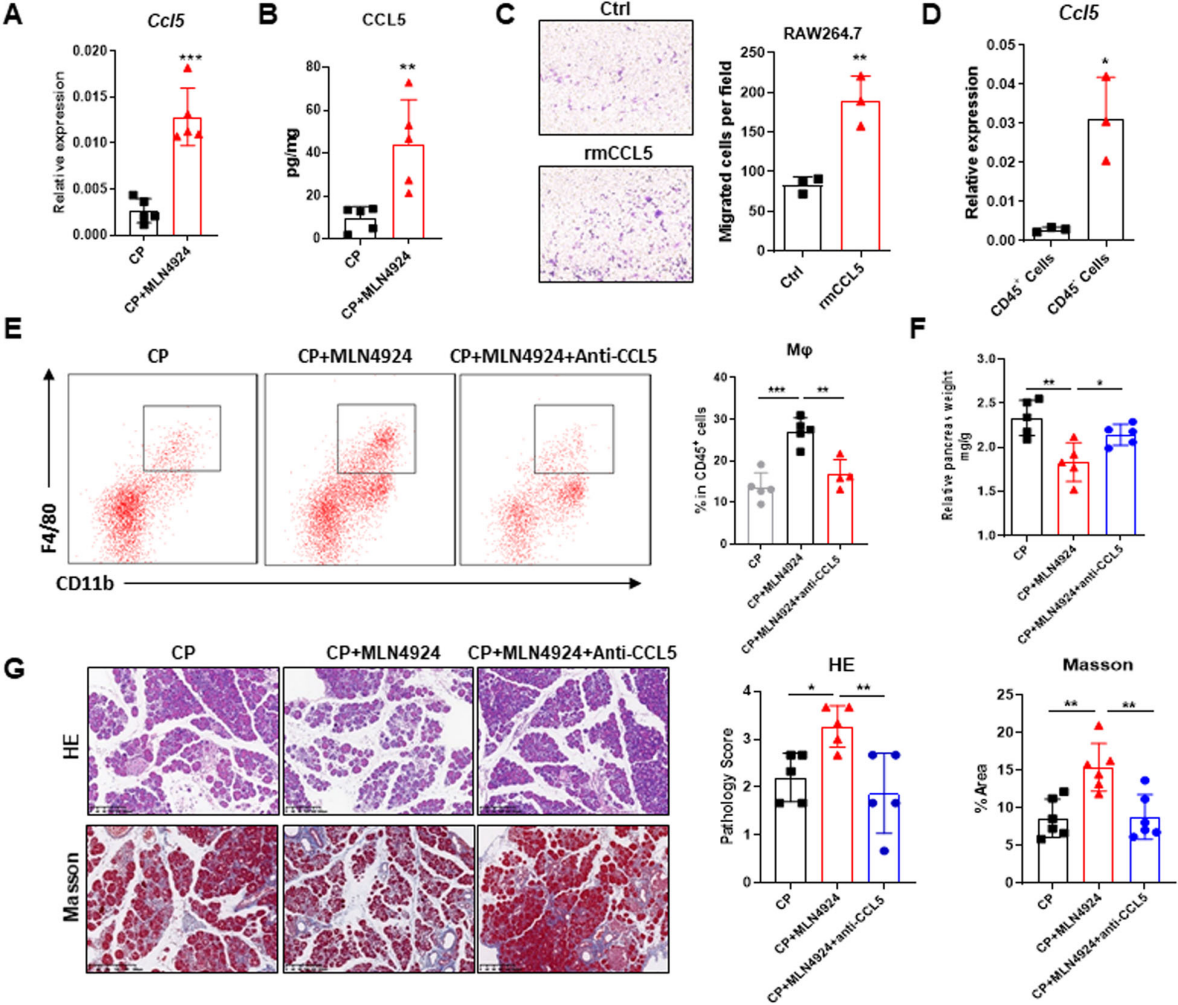
Neddylation inactivation elevates CCL5 secretion to recruit macrophages. **A** mRNA expression of *Ccl5* in pancreatic tissues. **B** Protein level of CCL5 in pancreatic lysate supernatants. **C** The migratory ability of rmCCL5-treated RAW264.7 cells, and the bar graph of migrated cells per field. *n* = 3. **D** mRNA expression of *Ccl5* in CD45^+^ and CD45^−^ cells sorted from the pancreata of MLN4924-treated CP mice. *n* = 3. **E** Representative flow cytometric figures and percentages of macrophages. **F** Relative pancreas weight (pancreas weight/body weight). **G** Representative H&E and Masson images of pancreata, and the bar graphs. Scale bar, 200 μm. **A**, **B**, **E**–**G** *n* = 5. **C**, **D** *n* = 3. Data are representative of three independent experiments. *p < 0.05, **p < 0.01, ***p < 0.001, ns no significant.

In view of the significant increase of macrophage infiltration, we were becoming inquisitive about the contributor to neddylation-regulated macrophage infiltration. We first examined the changes of key monocyte/macrophage recruitment factors, including CCL2, CCL5, CXCL12, and CSF1, in CP tissues after MLN4924 treatment^30,31^. Previous studies showed that CCL2 deficiency attenuated macrophage infiltration in CP mice^32^. Interestingly, we failed to discover significant changes in expression of *Ccl2* and other chemokines, such as *Cxcl12* and *Csf1*, at transcription level after MLN4924 treatment (Fig. S2). However, the mRNA expression of monocyte/macrophage chemokine *Ccl5* was significantly upregulated in MLN4924-treated CP mice compared with CP mice (Fig. 4A). Consistently, the protein level of CCL5 in pancreatic tissue was also significantly increased in MLN4924-treated CP mice (Fig. 4B). Moreover, RAW264.7 cells stimulated with rmCCL5 exhibited enhanced migratory ability (Fig. 4C), suggesting that CCL5 may be responsible for macrophage infiltration in CP. We distinguished pancreatic leukocytes with other pancreatic cells via CD45 expression and found that MLN4924-enhanced expression of *Ccl5* in CP mice was mainly derived from CD45^−^ cells (Fig. 4D), suggesting that CCL5 from non-hematopoieticcells was responsible for the increased infiltration of macrophages in CP microenvironment.

To evaluate the promoting roles of CCL5 on MLN4924-mediated CP, we neutralized CCL5 by anti-CCL5 antibody. CCL5 blockade caused a moderate reduction in the frequency of macrophages upregulated by MLN4924 in CP (Fig. 4E). The relative pancreas sizes of CP mice recovered to a certain extent in the presence of CCL5 neutralizing antibody (Fig. 4F). Moreover, histological analysis exhibited relatively normal tissue architecture and less infiltrated immune cells via CCL5 blockade (Fig. 4G). Masson trichrome gelatin staining of pancreata demonstrated that CCL5 blockade impaired fibrosis to a certain extent (Fig. 4G). Collectively, CCL5 is required for MLN4924-mediated macrophage infiltration in chronic pancreatitis.

### Neddylation inactivation promotes CCL5 secretion in a HIF-1α dependent manner

**Fig. 5:**
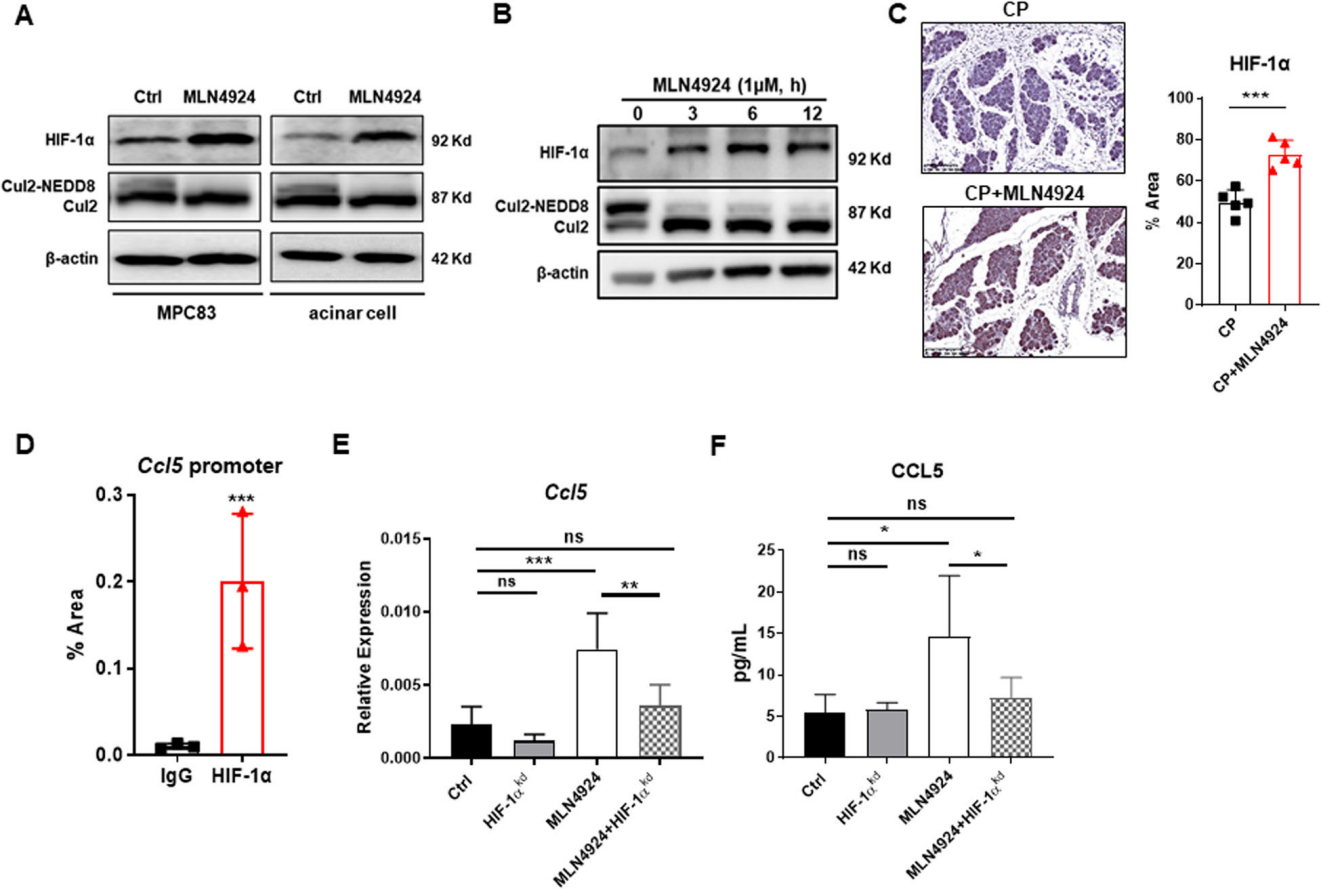
Neddylation inactivation upregulates CCL5 via a HIF-1α dependent manner. **A** WB analysis of HIF-1α and cullin 2 in MPC-83 cells and primary acinar cells. **B** WB analysis of HIF-1α and cullin 2 in pancreatic acinar cells. **C** Representative IHC images of HIF-1α, and bar graphs. Scale bar, 200 μm. **D** ChIP-qPCR analysis of HIF-1α binding to the *Ccl5* promoter region, IgG was used as the control. **E** mRNA expression of *Ccl5* in si-*Hif1a* or si-*scramble* treated acinar cells. **F** Secretion levels of CCL5 in si-*Hif1a* or si-*scramble* treated acinar cells. **A**, **B**, **D** *n* = 3. **C**, **E**, **F** *n* = 5. Data are representative of three independent experiments. *p < 0.05, **p < 0.01, ***p < 0.001, ns no significant. Cul-2, cullin2, pos positive control, neg negative control.

To investigate the mechanisms that neddylation regulates CCL5 secretion to direct macrophage infiltration in CP, we were suggested to look for functional substrates of neddylation. We detected the substrate molecules of neddylation, such as HIF-1α, c-myc and c-Jun, which can regulate the transcription of CCL5. Murine acinar cells isolated from pancreata and pancreatic acinar cell line MPC-83 were stimulated with MLN4924 for 12 h. We found that MLN4924 could not change the protein levels of c-myc and c-Jun (Fig. S3). HIF-1α, a well-known substrate of CRL2^VHL^, is stabilized and translocated to the nucleus for enhancing transcription of numerous inflammatory genes in inflammation and tumor^33,34^. We found an upregulated protein level of HIF-1α and reduced radio of NEDD8-conjugated to unconjugated cullin 2 in MLN4924-treated group, indicating the inhibited neddylation status and the accumulated HIF-1α (Fig. 5A). Moreover, MLN4924 induced accumulation of HIF-1α in pancreatic acinar cells with time dependence (Fig. 5B). Immunohistochemistry further confirmed greater expression of HIF-1α in pancreatic acinus of MLN4924-treated CP mice (Fig. 5C). Hence, neddylation inactivation contributed to HIF-1α accumulation in pancreatic acinar cells. To gain further insight into the transcriptional regulation of HIF-1α on *Ccl5*, we performed a Chip assay and ascertained that HIF-1α directly binds to the promotor region of *Ccl5* in pancreatic acinar cells (Fig. 5D). Next, we interfered the expression of HIF-1α in pancreatic acinar cells and the knockdown efficiency was detected by western blot (Fig. S4). si-*Hif1a* treated cells showed a significant decrease in CCL5 expression at gene transcription and translation levels (Fig. 5E, F). Thus, HIF-1α acts as a potent modulator of neddylation-induced CCL5 expression in chronic pancreatitis.

### Expression profile in chronic pancreatitis patients and working model

**Fig. 6:**
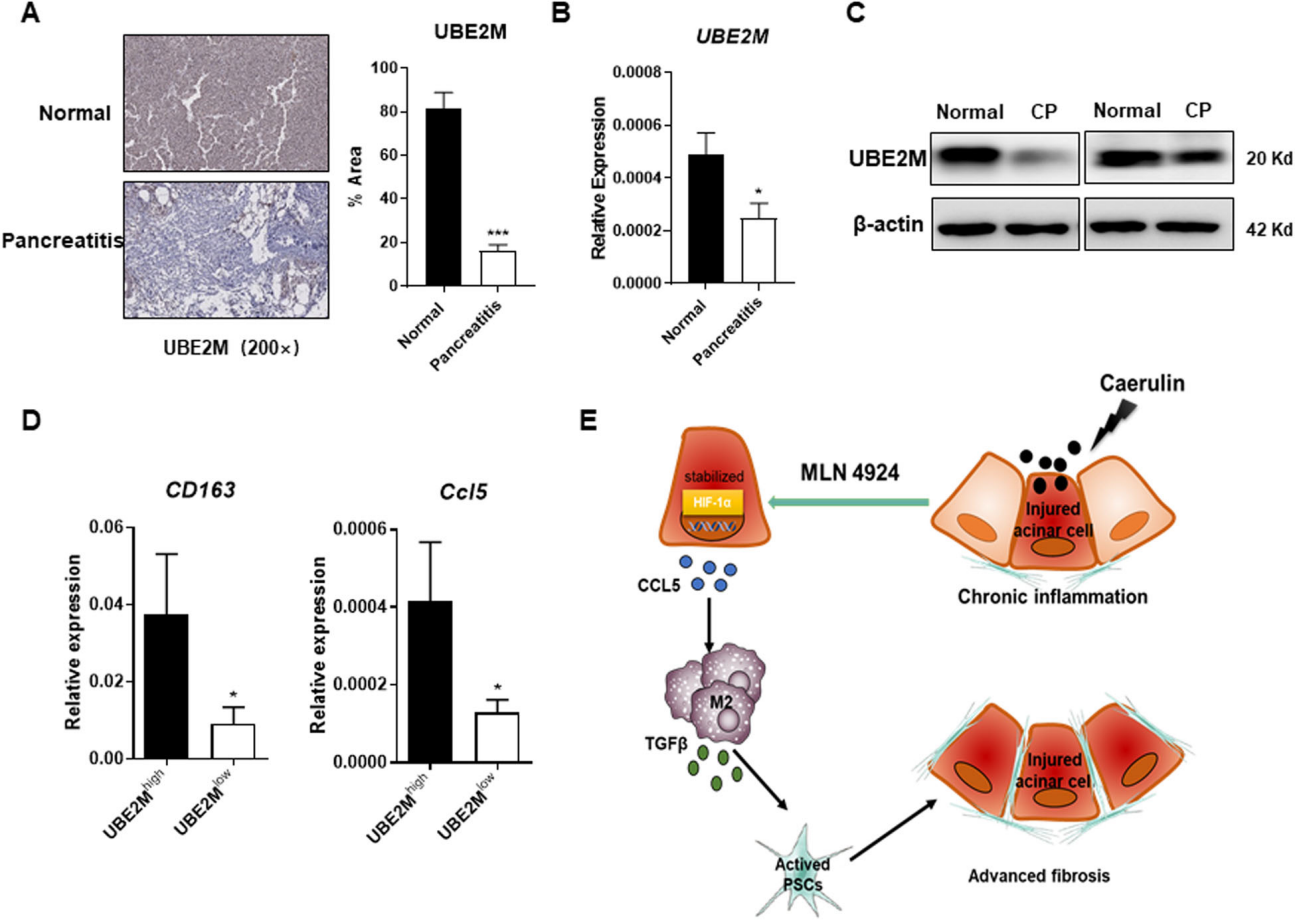
Expression profile in CP patients and working model. **A** Representative IHC images of UBE2M expression in CP specimens and normal tissues, and the bar graphs. **B** mRNA expression of *UBE2M* in CP specimens and normal tissues. **C** Western blot analysis of UBE2M in CP specimens and normal tissues. **D** mRNA expression of *CCL5* and *CD163* in UBE2M^high^ and UBE2M^low^ CP specimens. **E** A working model depicting that inactivation of neddylation pathway by MLN4924 stabilizes HIF-1α level, upregulates CCL5 expression, accelerates macrophage infiltration, and eventually exacerbates chronic pancreatitis. **A**–**D** *n* = 3. Data are representative of three independent experiments. *p < 0.05, **p < 0.01, ***p < 0.001, ns no significant.

By far, we have determined that global inactivation of the neddylation pathway played a promoting role in the immunopathogenesis of CP, which suggesting that maintaining the activation of neddylation may represent an effective therapeutic strategy for CP. Next, we made attempts to extend our explorations in the murine model to human CP patients. To this end, we collected pancreatic specimens from patients with clinically confirmed chronic pancreatitis and analyzed the expression of UBE2M by immunohistochemistry, RT-PCR and western blot. Data showed that the protein level of UBE2M was lower in CP specimens compared to corresponding normal tissues (Fig. 6A). The CP specimens also had much lower mRNA level of *UBE2M* than normal tissues (Fig. 6B). According to the detected expression intensity of UBE2M, we divided the CP specimens into two groups. As we anticipated, the mRNA expression levels of *CCL5* and M2 marker *CD163* were negatively correlated with the protein level of UBE2M (Fig. 6D), suggesting the neddylation-associated modulation of macrophages in chronic pancreatitis. In summary, our studies reveal a neddylation/HIF-1α/CCL5 axis in regulating macrophage infiltration in chronic pancreatitis and further represent new ideas for therapeutic strategy and disease treatment (Fig. 6E).

## Discussion

Despite advances in understanding the pathogenesis of chronic pancreatitis, much more remains to be done. In this study, we use a model of chronic pancreatitis induced by caerulein, which is widely used and the best characterized experimental model^35,36^, to investigate the pathogenesis of pancreatitis. Our findings provide several lines of evidence for the important roles of neddylation in regulating the immunopathogenesis of chronic pancreatitis. We found that the expression of the NEDD8-conjugating enzymes significantly decreased in CP pancreata and neddylation protects against pancreatitis. Moreover, inactivation of neddylation promotes the CCL5-dependent infiltration of M2 macrophages into the inflammatory pancreata, which dependent on HIF-1α increased CCL5 secretion. Together, this study reveals the immune mechanisms of neddylation in chronic pancreatitis and provides potential targets for immune-based therapies.

Neddylation acts as an important mediator in regulating the inflammation and inactivation of neddylation usually exhibits beneficial effects on disease control. Inactivation of neddylation has been shown to repress LPS-induced proinflammatory cytokine production through restriction of the CRL/NF-κB signal and alleviate sepsis-induced acute kidney injury and acute lung injury^29,37^. Neddylation inactivation also exhibits its pronounced anti-viral efficacy and anti-inflammation activity by promoting type I interferon production through CRLs-dependent mechanisms^38^. Additionally, diminished neddylation activity via MLN4924 could be important for resolving liver fibrosis and pulmonary fibrosis^39,40^. However, in contrast to our current knowledge, our study reveals an opposite effect of MLN4924 in regulating caerulein-induced chronic pancreatitis. Global inactivation of neddylation by MLN4924 leads to HIF-1α accumulation in acinus and promotes secretion of CCL5, which further recruits M2 macrophages to pancreata and accelerates fibrosis and CP. This suggests that the role of neddylation on inflammation may be organ or microenvironment dependent.

As demonstrated by previous studies, the immune characteristic of CP is infiltration with numerous macrophages^8,9^. Once activated by various signals in the microenvironment, macrophages produce TGF-β and other fibrogenic factors. This process exacerbates fibrosis and chronic pancreatitis, but the detailed mechanism remains confused^41^. Neddylation inactivation can diminish macrophage function via regulating the activation of inflammasome and the secretion of proinflammatory cytokines^24,42,43^. Moreover, neddylation inactivation affects the viability of macrophages^44^. All the studies mentioned put their eyes on the modulation of MLN4924 on macrophage function, how it influences the chemotaxis of macrophages remains unknown. Our colleagues previously discovered that neddylation inactivation could suppress the recruitment of TAMs in lung tumor^45^, indicating that the MLN4924 could regulate macrophage accumulation. It is reported that CP tissues from patients have increased CCL5, suggesting the potential pathogenic roles for CCL5 in CP^10^. In this study, we demonstrate the acinar cell-modulated recruitment of macrophages via CCL5 and show an important role of neddylation inactivation by MLN4924 in a crosstalk between macrophages and acinar cells in pancreata. However, the paradoxical phenomenon of MLN4924-regulated chemotaxis is observed in different animal models, we speculate that the disparate pathogenesis and novel CRL-substrates regulating chemokine expression need to be further concerned.

Collectively, our investigation demonstrates that neddylation plays an important role in regulating the immune microenvironment during pancreatitis. Given the protective effect of neddylation in the process of CP, MLN may not be suitable for treating pancreatic disease and we should try to maintain or even improve the level of neddylation to prevent pancreatitis.

## Materials and methods

### Mice and treatments

All procedures were performed with the approval of the Institutional Animal Care and Use Committee of Fudan University. 6-week-old female C57BL/6 mice were purchased from SIPPC-BK Laboratory Animal Co. Ltd. C57BL/6 mice of the same sex and similar age, which were obtained from the same vendor, were randomly allocated to the experimental conditions and treatment groups. All mice were bred and maintained in specific-pathogen-free (SPF) conditions. Chronic pancreatitis was induced by repetitive caerulein injections^36,46^. Briefly, mice were administered six-hourly intraperitoneal (i.p.) injections of 50 μg/kg body weight caerulein (MCE) 3 days per week, for a total of 4 weeks. Mice were sacrificed 3 days after the last injection. For neddylation inactivation, mice were subcutaneously administrated of 60 mg/kg MLN4924 once a day, on a 5-days-on/2-days-off schedule for 4 cycles. Clodronate liposomes (FormuMax Scientific) were used for macrophage depletion. Mice were injected intraperitoneally with 100 μL liposomes twice a week for 4 weeks. For neutralizing experiments, mice were treated i.p. with 2 μg αCCL5 (R&D system) 3 times a week for 4 weeks. For mouse experiments, sample sizes were rationalized by balancing sufficient replication to detect significant differences between groups with reduction of total animals used. The animals were kept in a specific-pathogen-free environment. All animal experiments were approved by the Institution of Animal Care and Use Committee of Fudan University.

### Histology, immunohistochemistry, and immunofluorescence

Immunohistochemistry was performed using the avidin-biotin-peroxidase complex method. The pathological processes were scored as previously described^47^, according to the tissue architecture, the glandular atrophy and the infiltration of immune cells. The scoring of Masson trichrome gelatin staining was based on the percentage of collagen fibers. For immunofluorescence, the slides were incubated with antibodies overnight, followed by fluorescently-labeled antibody incubation. Images were acquired with confocal microscopes (Leica). The antibodies used were listed in Supplementary Table S1.

### Cell isolation, purification, and flow cytometry

Pancreatic tissues were digested with 1 mg/mL collagenase IV to prepare single-cell suspensions. Single-cell suspensions were stained with different antibodies listed in Supplementary Table S2. The data were acquired on a BD Celesta instrument and analyzed with FlowJo software (San Carlos). For cell purification, suspensions were stained and sorted by flow cytometry (Melody, BD). The purity of sorted cells was >99%. Murine pancreatic acinar cells were isolated as previous described^48^.

### Quantitative RT-PCR, western blotting, and enzyme-linked immunosorbent assay

RT-PCR was performed with a QuantStudio™ 5 PCR system (ABI). The relative expression of genes was calculated using the 2^−ΔΔCt^ method. The primer sequences are shown in Supplementary Table S3. Protein concentration was quantified using BCA assay Kit (Pierce Biotechnology). The antibodies used for western blotting were listed in Supplementary Table S4. Cultural supernatants and tissue lysate supernatants were analyzed using enzyme-linked immunosorbent assay kit for mouse CCL5 detection (R&D system).

### Chemotaxis assays

The standard chemotaxis assay was performed using a transwell polycarbonate filter (8-μm pore size; Corning). 2 × 10^4^ cells were plated in the upper chambers, and the lower chamber contained DMEM with 100 ng/mL recombinant murine CCL5 (rmCCL5, PeproTech). After incubation for 12 h, migrated cells were fixed in 4% paraformaldehyde and stained with 0.1% crystal violet for 30 min, followed by photographing under a general microscope.

### Cell culture, treatment, and siRNA transfection

Murine pancreatic acinar cell line MPC-83 was purchased from Procell Biotechnology Company and cultured in the recommended medium. The cell line has been validated by the supplier and further tested for mycoplasma contamination in our laboratory. Cells were treated with 1 μM MLN4924 for indicated times. Specific siRNA targeting *Hif1a* and scrambled siRNA were synthesized by GenePharma. The si-*Hif1a* sequences were: CCAUGUGACCAUGAGGAAATT. Lipofectamine^®^ RNAiMAX reagent (Invitrogen) was added to perform siRNA transfection according to the manufacturer’s protocol.

### Chromatin immunoprecipitation (ChIP)

The experiments in this paper were performed by using SimpleChIP^®^ Plus Enzymatic Chromatin IP Kit (CST) according to the manufacturer’s instructions. Fragmented chromatin was incubated with anti-HIF1α antibody (CST) or control antibodies applied in the ChIP kit. Immunoprecipitated DNA was purified, amplified, and separated by electrophoresis. The primers targeting the promoter of *Ccl5* were: 5’-CACTACAGTTGGCTTCCGGT-3’ and 5’-AAAGTTTAGGGGGCATCGGG-3’.

### Human specimens

6 human CP specimens and 3 normal pancreatic specimens were obtained from Ren Ji Hospital from January 2018 to January 2020. CP patients had clinical features, such as chronic or relapsing pancreatic-type pain, and confirmed pathological diagnosis by computed tomography scan and ultrasound. Normal specimens were collected from the adjacent pancreatic tissues of duodenal neuroendocrine tumor (G1). All the patients were provided with written informed consent and the study was approved by the Research Ethics Committee of Ren Ji Hospital, School of Medicine, Shanghai Jiao Tong University.

### Statistical analysis

Statistical analyses were performed using Prism Graph 7 software. All data were presented as mean ± SEM. Two-tailed Student’s *t* test was used for comparisons between two groups. Multiple-group comparisons were performed using one-way ANOVA followed by Tukey’s multiple comparisons test. *p* < *0.05* were considered statistically significant.

## Supplementary information

Supplementary Figure

Supplementary Tables
