# Supplementary material for: Neddylation pathway alleviates chronic pancreatitis by reducing HIF1α-CCL5-dependent macrophage infiltration
Source: Cell Death Dis. 2021 Mar 15;12(3):273. doi: 10.1038/s41419-021-03549-3 (PMC7960984; doi:10.1038/s41419-021-03549-3)
Supplement: Supplementary file 1 — Supplementary Figure [file 41419_2021_3549_MOESM1_ESM.pptx]

## Slide 1
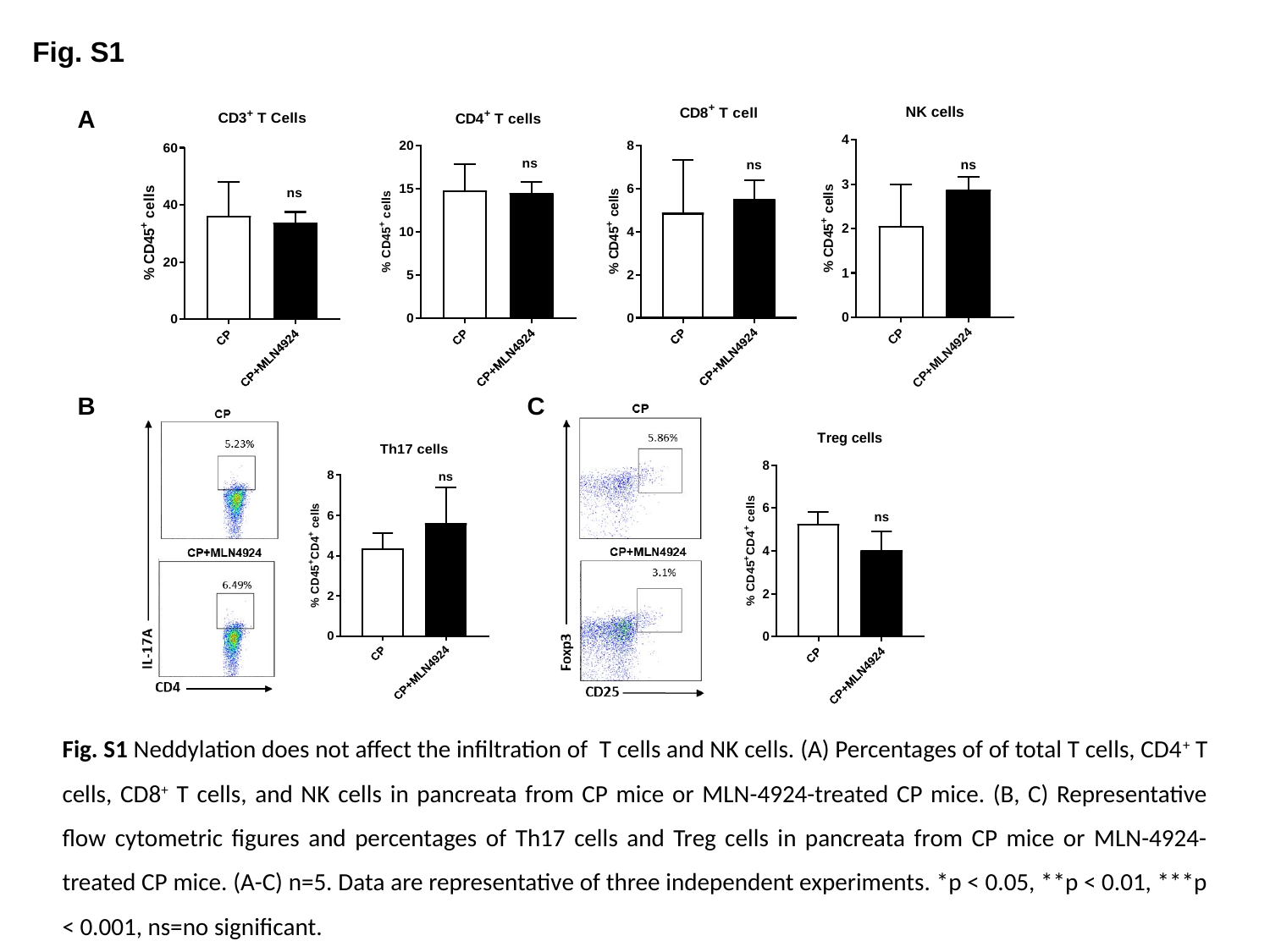

Fig. S1
A
C
B
Fig. S1 Neddylation does not affect the infiltration of T cells and NK cells. (A) Percentages of of total T cells, CD4+ T cells, CD8+ T cells, and NK cells in pancreata from CP mice or MLN-4924-treated CP mice. (B, C) Representative flow cytometric figures and percentages of Th17 cells and Treg cells in pancreata from CP mice or MLN-4924-treated CP mice. (A-C) n=5. Data are representative of three independent experiments. *p < 0.05, **p < 0.01, ***p < 0.001, ns=no significant.

## Slide 2
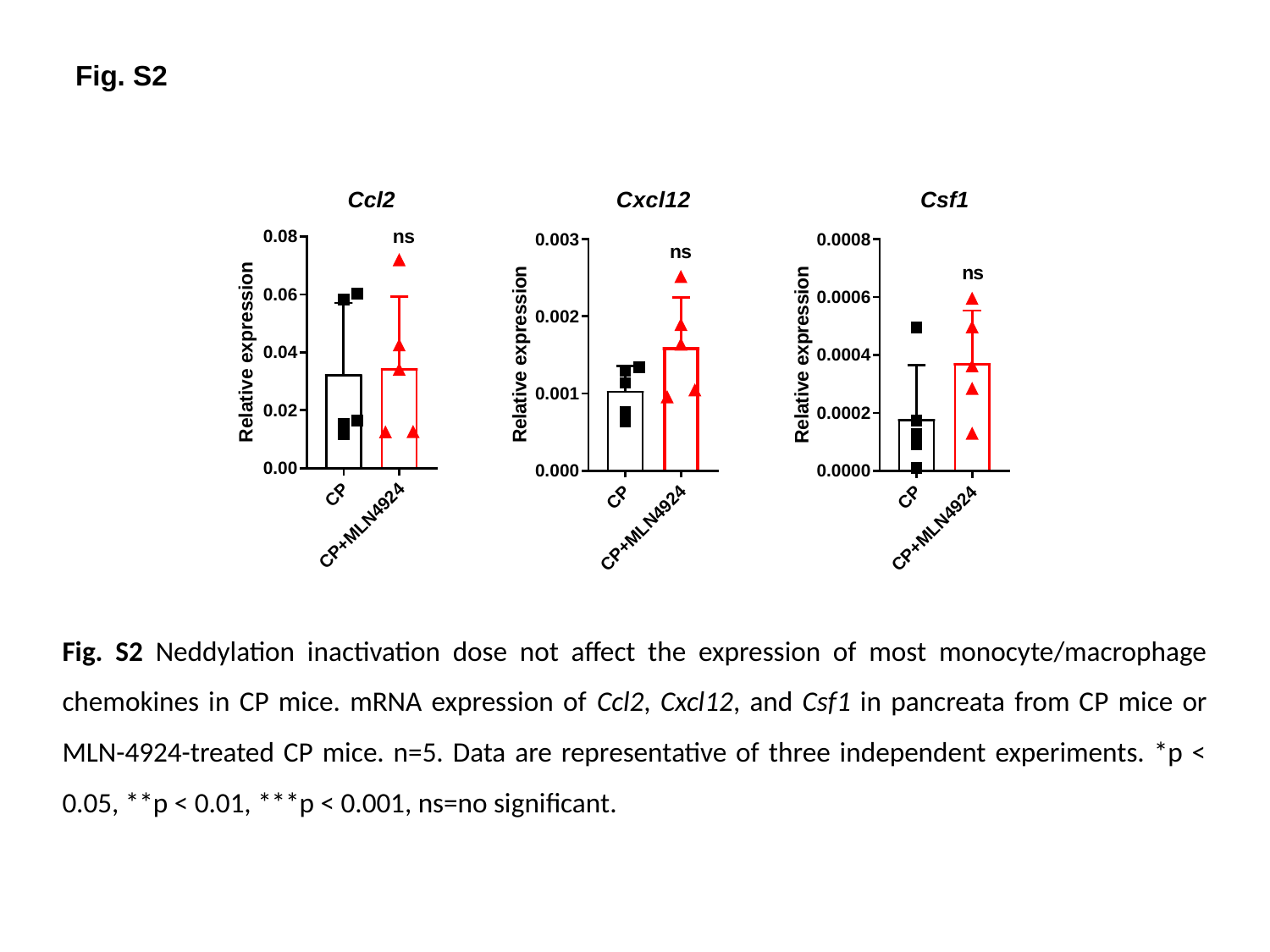

Fig. S2
Fig. S2 Neddylation inactivation dose not affect the expression of most monocyte/macrophage chemokines in CP mice. mRNA expression of Ccl2, Cxcl12, and Csf1 in pancreata from CP mice or MLN-4924-treated CP mice. n=5. Data are representative of three independent experiments. *p < 0.05, **p < 0.01, ***p < 0.001, ns=no significant.

## Slide 3
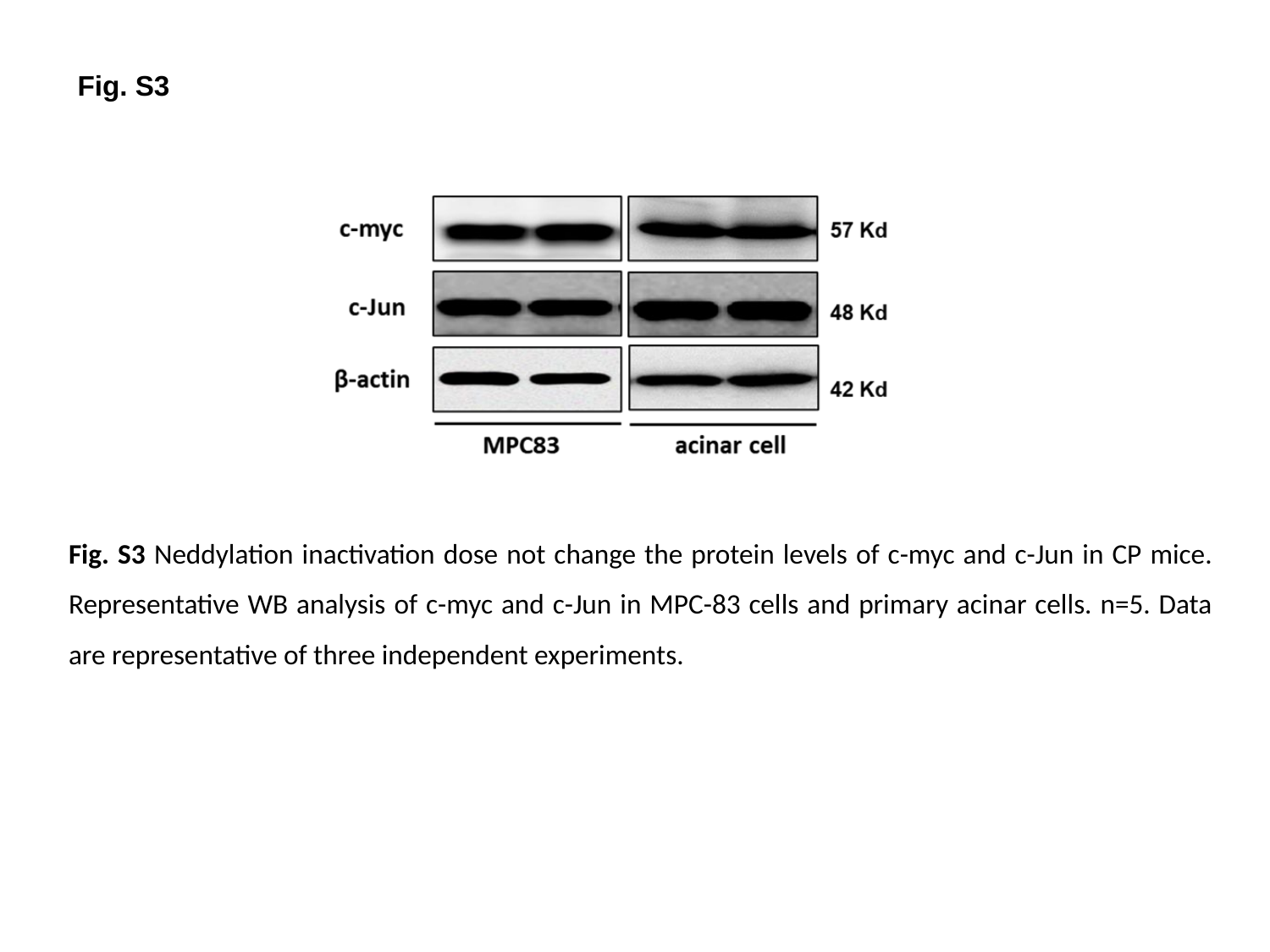

Fig. S3
Fig. S3 Neddylation inactivation dose not change the protein levels of c-myc and c-Jun in CP mice. Representative WB analysis of c-myc and c-Jun in MPC-83 cells and primary acinar cells. n=5. Data are representative of three independent experiments.

## Slide 4
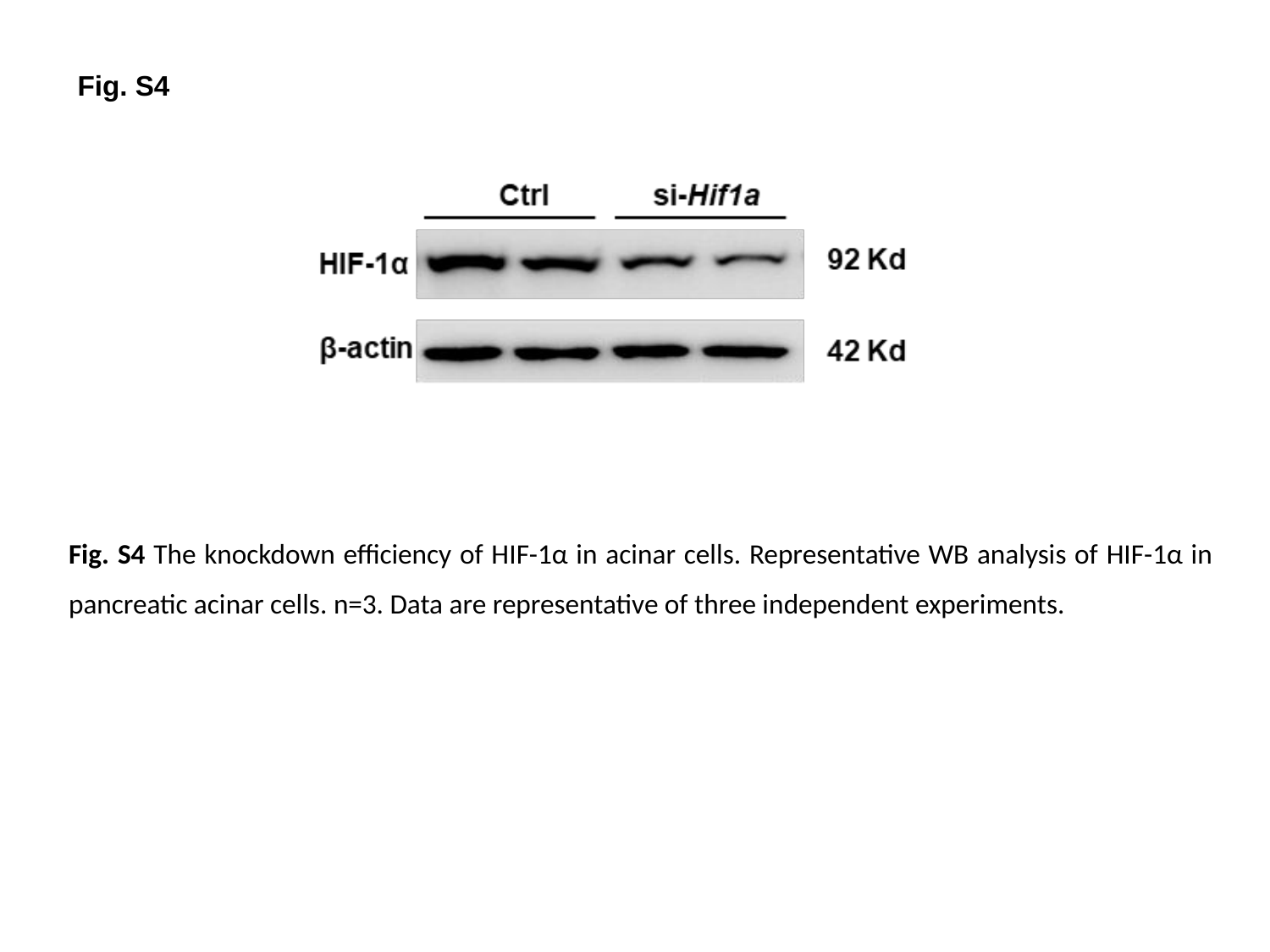

Fig. S4
Fig. S4 The knockdown efficiency of HIF-1α in acinar cells. Representative WB analysis of HIF-1α in pancreatic acinar cells. n=3. Data are representative of three independent experiments.
