# Supplementary material for: Neddylation pathway alleviates chronic pancreatitis by reducing HIF1α-CCL5-dependent macrophage infiltration
Source: Cell Death Dis. 2021 Mar 15;12(3):273. doi: 10.1038/s41419-021-03549-3 (PMC7960984; doi:10.1038/s41419-021-03549-3)
Supplement: Supplementary file 2 — Supplementary Tables [file 41419_2021_3549_MOESM2_ESM.docx]

**Table S1.** **The antibody used for Immunohistochemistry and Immunofluorescence**

| Antibody | Dilution | Catlog Number | Company |
| --- | --- | --- | --- |
| UBE2M | 1:1000 | 109507 | abcam |
| Collagen I | 1:500 | GB11022-1 | Service bio |
| HIF-1α | 1:200 | 14179 | abcam |
| F4/80 | 1:500 | GB11027 | Service bio |
| iNOS | 1:500 | GB11119 | Service bio |

**Table S2.** **The antibody used for flow cytometer**

|  | Clone | Company |
| --- | --- | --- |
| Anti-Mouse CD11b | M1/70 | BD Biosciences |
| Anti-Mouse F4/80 | T45-2342 | BD Biosciences |
| Anti-Mouse CD45 | 30-F11 | BD Biosciences |
| Anti-Mouse CD206 | MMR | Biolegend |

**Table S3.**  **The primer sequences of genes for RT-PCR**

| **Murine** | **Forward** | **Reverse** |
| --- | --- | --- |
| *Actb* | TGTCCACCTTCCAGCAGATGT | AGCTCAGTAACAGTCCGCCTAGA |
| *Ube2m* | AACCTGCCCAAGACGTGTG | AGCTGAATACAAACTTGCCACT |
| *Ube2f* | ACGCTGGCAAGCAAGTTGA | CCTCATCTGGGCTTACAGTCAG |
| *Tgfb1* | CTCCCGTGGCTTCTAGTGC | GCCTTAGTTTGGACAGGATCTG |
| *Acta2* | TCCTGACGCTGAAGTATCCGATA | GGCCACACGAAGCTCGTTAT |
| *Arg1* | CTCCAAGCCAAAGTCCTTAGAG | AGGAGCTGTCATTAGGGACATC |
| *Mrc1* | CTCTGTTCAGCTATTGGACGC | CGGAATTTCTGGGATTCAGCTTC |
| *Retnla* | CCAATCCAGCTAACTATCCCTCC | ACCCAGTAGCAGTCATCCCA |
| *Nos2* | GTTCTCAGCCCAACAATACAAGA | GTGGACGGGTCGATGTCAC |
| *Tnfa* | CTGAACTTCGGGGTGATCGG | GGCTTGTCACTCGAATTTTGAGA |
| *Ccl2* | TTAAAAACCTGGATCGGAACCAA | GCATTAGCTTCAGATTTACGGGT |
| *Ccl5* | GCTGCTTTGCCTACCTCTCC | TCGAGTGACAAACACGACTGC |
| *H-2b* | GACCACGTAGGCACCTATGG | CTACAGCTATGTTTTGCAGTCCA |
| **Human** | **Forward** | **Reverse** |
| *Ube2m* | ATGAGGGCTTCTACAAGAGTGG | ATTGTCTCACACTTCACCTTGG |
| *Ccl5* | ACCAGTGGCAAGTGCTCCA | ACCCATTTCTTCTCTGAGTTGGCA |
| *Cd163* | TTTGTCAACTTGAGTCCCTTCAC | TCCCGCTACACTTGTTTTCAC |

**Table S4. The antibody used for Western blotting**

| Antibody | Dilution | Catlog Number | Brand |
| --- | --- | --- | --- |
| β-Actin | 1:5000 | 4970 | Cell Signaling Technology |
| HIF-1α | 1:1000 | 14179 | Cell Signaling Technology |
| Cullin 2 | 1:1000 | 166917 | Abcam |
